# From data mining to experimental validation: ST36 and CV12 as core acupoint combination for acupuncture in functional dyspepsia

**DOI:** 10.3389/fmed.2026.1728742

**Published:** 2026-03-10

**Authors:** Jialin Jia, Zhen Zhong, Tie Li, Min He, Mengmeng Sun, Jing He, Xingbang Wang, Ru Nie, Jiazhen Cao, Guojuan Dong

**Affiliations:** 1 Department of Acupuncture and Tuina, Changchun University of Chinese Medicine, Changchun, China; 2 Northeast Asia Research Institute of Traditional Chinese Medicine, Changchun University of Chinese Medicine, Changchun, China; 3 Department of Nursing, Changchun University of Chinese Medicine, Changchun, China

**Keywords:** acupoint prescription, acupuncture, association analysis, data mining, duodenal low-grade inflammation, functional dyspepsia

## Abstract

**Background:**

Effective treatments for functional dyspepsia (FD) remain elusive. Acupuncture presents promising potential due to its safety and minimal side effects. However, variability in acupoint prescriptions in clinical practice has hindered the optimization and dissemination of treatment protocols for FD. This study seeks to screen out the core acupoint combination, and experimentally validate its efficacy.

**Methods:**

This study involved searching eight databases to analyze acupoint prescriptions in the literature using data mining and association rules. The aim was to identify an acupoint combination based on prescription frequency, co-occurrence patterns, and network relevance as a core prescription. Subsequently, we established the FD rat model. The rats were randomly assigned to control, model, electroacupuncture, and Itopride groups (*n* = 6 per group). All interventions lasted 14 days. The body weight, 3 h food intake, and gastric emptying rate were assessed in each group. Eosinophil (Eos) and mast cell (MC) counts in duodenal tissues were determined using Eos staining and toluidine blue staining, respectively. Pathological changes in duodenal tissues were examined through hematoxylin–eosin (H&E) staining, while immunofluorescence (IF) staining was employed to evaluate the expression levels of tight junction proteins Claudin-1 and Occludin in the duodenal mucosal barrier.

**Results:**

The data mining results revealed ST36 as the most frequently utilized acupoint, with its combination with CV12 being the most prevalent. Network association analysis showed the interconnections among them. Integrating multiple analytical methods, this study ultimately suggests the central role of ST36 and CV12 in acupoint prescriptions for the treatment of FD. Animal experiments demonstrated that electroacupuncture stimulation of these two acupoints significantly improved digestive function and nutritional status in FD rats. Eos staining and toluidine blue staining indicated a marked reduction in Eos and MC infiltration. Histological examination showed restoration of duodenal villi structure, while IF further showed increased expression of Claudin-1 and Occludin.

**Conclusion:**

This study showed that ST36 and CV12 are the core acupoint combination of acupuncture for FD. Animal experiments further demonstrated that electroacupuncture stimulation of these two acupoints could significantly alleviate the duodenal low-grade inflammation and mucosal barrier damage in FD rats.

## 1 Introduction

Functional dyspepsia (FD) is a prevalent chronic functional gastrointestinal disorder. It is characterized by symptoms such as postprandial fullness, early satiety, epigastric pain, and epigastric burning sensation (1). The global prevalence of FD is approximately 7% and continues to grow (2, 3). Although FD does not directly affect mortality (4, 5), it significantly impairs patients’ quality of life and imposes a substantial economic burden (6). The pathogenesis of FD has not been fully elucidated, though it is associated with visceral hypersensitivity, gastrointestinal motility disorders, and brain-gut axis dysfunction (1, 7). According to the treatment guidelines of various countries (8–13), FD treatment remains based on symptomatic management. Common pharmacological treatments include proton pump inhibitors, histamine-2 receptor antagonists, prokinetic agents, and central neuromodulators (1, 14). However, these approaches have limited efficacy and carry risks of side effects and adverse reactions. Consequently, the exploration of efficient and safe alternative therapies has become an important research direction in this field.

Acupuncture is a traditional external therapy known for its holistic effects and minimal side effects. It is particularly suitable for FD, which has a complex etiology. Numerous studies have demonstrated its significant efficacy in functional gastrointestinal disorders (15–17). Although numerous studies have preliminarily summarized the patterns of acupoint usage in acupuncture for FD (18–20), rigid standardization may violate the inherent principle of individualization in Chinese medicine, thereby limiting the clinical applicability and translational value of the research findings. This study aims to explore a reproducible minimal core framework (a set of highly recurrent and structurally central acupoints) in acupuncture prescriptions for FD by analyzing existing literature. This prescription can serve as a reference for clinicians while also allowing for individualized adjustments based on syndrome differentiation and patient characteristics. This approach achieves an effective balance between standardization and individualization, providing a foundation for optimizing clinical treatment protocols. Furthermore, methodologically, compared to previous studies, we integrate frequency analysis, association rule mining, and network-based centrality/k-core analysis to characterize the highly recurrent combinations and structural hubs within the prescriptions used in FD randomized controlled trials (RCTs).

To validate the treatment relevance of the core acupoint combination, this study conducted additional animal experiments. Recent research has highlighted the correlation between the pathogenesis of FD and duodenal low-grade inflammation (21, 22), characterized by eosinophil (Eos) and mast cell (MC) infiltration (23–25). Additionally, impaired integrity of the duodenal mucosa and decreased expression of inter-epithelial tight junction proteins (TJs) are also associated with this inflammation (26–28). Researchers hope to find new therapeutic targets for this pathology as a breakthrough for the future treatment of FD (29). However, effective interventions for these pathological changes remain elusive. Therefore, this study used these pathological changes as the main indicators to evaluate the therapeutic effects of electroacupuncture stimulation on core acupoints in FD rats.

In summary, this study integrates literature analysis with experimental validation. Initially, data mining and network correlation analysis summarized ST36 and CV12 as the core acupoint prescription for acupuncture treatment of FD. Then, through Eos staining, toluidine blue staining, and IF staining, subsequent experiments demonstrated the therapeutic effects of electroacupuncture stimulation of these acupoints on low-grade inflammation and barrier damage in the duodenum of FD rats. Our findings not only demonstrated the key role of the combined use of ST36 and CV12 in the acupuncture treatment of FD, but also provided valuable evidence that acupuncture modulates duodenal low-grade inflammation in FD.

## 2 Materials and methods

### 2.1 Literature search strategy

We searched four international electronic databases: PubMed, the Cochrane Library, Embase, and Web of Science, as well as four Chinese databases: China National Knowledge Infrastructure, Wanfang Database, VIP Database, and Chinese Biomedical Database. The search scope covered all literature published from the establishment of each database to June 30, 2025. We did not apply language restrictions at the search stage. During full-text screening, studies were eligible if an English or Chinese full text could be obtained; records in other languages were documented and the reasons for exclusion were recorded in the PRISMA flow diagram. The customized search strategies for each database are detailed in Supplementary material 1. To ensure the completeness of the literature, we also conducted a secondary screening of relevant systematic reviews and references of the identified studies.

### 2.2 Inclusion and exclusion criteria

The inclusion criteria were as follows: ① Participants: patients diagnosed with FD using recognized diagnostic criteria (e.g., Rome I/II/III/IV or national/clinical consensus guidelines) (30–33). ② Intervention: needle-based acupuncture as the primary intervention (manual acupuncture and/or electroacupuncture). Non-penetrating or non-needle acupoint stimulation (e.g., laser acupuncture), auricular/scalp acupuncture, warm-needle acupuncture, acupoint injection, acupoint patches, and moxibustion were excluded unless clearly specified and justified *a priori*. ③ Comparison: sham acupuncture, recommended pharmacotherapy (e.g., prokinetics), usual care, waitlist, or no-treatment controls. ④ Outcomes: the trial reported at least one clinically relevant FD outcome (e.g., Nepean Dyspepsia Index, gastrointestinal symptom scores) and/or safety outcomes; ⑤ Study type: randomized controlled trials.

The exclusion criteria were as follows: ① Studies enrolling participants with confirmed organic gastrointestinal diseases (e.g., peptic ulcer, malignancy, inflammatory bowel disease) or other major organic pathologies based on objective examinations (e.g., endoscopy, imaging, laboratory tests), unless FD was clearly diagnosed separately; ② Studies focusing primarily on non-needle acupoint interventions (e.g., acupoint patches, acupoint injection, moxibustion, acupressure); ③ Non-randomized studies, observational studies, case reports, animal studies, protocols, or reviews; ④ Studies in which participants had clearly diagnosed psychiatric disorders (e.g., major depressive disorder, anxiety disorders) that could confound FD outcomes, based on medical diagnosis/recorded treatment, unless explicitly balanced across groups; ⑤ Studies with irretrievable full texts, insufficient information to extract acupoint prescriptions, or duplicate publications (in which case the most complete report was retained); ⑥ Studies published only as abstracts.

### 2.3 Data extraction

Literature was screened using EndNote X8 software. Each eligible study was extracted independently by two reviewers. Extracted information included author, year, country, diagnostic criteria, sample size, intervention details (including acupoint set, stimulation parameters, frequency/duration), comparator type, outcomes, and adverse events. Acupoint names were standardized according to the WHO Standard Acupuncture Point Locations in the Western Pacific Region (34). For example, “ZU Sanli” were mapped to the standardized code ST36. Acupoint prescription was defined as the complete set of acupoints used in the acupuncture treatment arm of an RCT. All standardized acupoint prescriptions were imported into SPSS Modeler 18 for analysis.

### 2.4 Quality assessment

The included studies were evaluated for quality using the Cochrane risk assessment tool, covering dimensions such as random sequence generation, allocation concealment, blinding of subjects and researchers, blinding of outcome assessment, incomplete outcome data, selective reporting, and other biases. Each indicator was classified as high risk, low risk, or unclear risk. Two reviewers (Zhen Zhong and Jialin Jia) assessed study quality independently, while a third reviewer (Jiazhen Cao) resolved any disagreements.

### 2.5 Data analysis

We first conducted a frequency analysis of the acupoints selected for treating FD to characterize their usage patterns. Next, we applied the Apriori algorithm in SPSS Modeler 18 to perform a correlation analysis, mining association rules among the commonly used acupoints based on pre-specified support and confidence thresholds. This approach identifies frequent co-occurrence patterns and structural relationships within the prescription dataset rather than directly measuring clinical efficacy, which better reflects the strengths and limitations of association rule mining in clinical data contexts. Association rule mining, including the use of the Apriori algorithm with user-defined thresholds such as support and confidence, is widely used in healthcare data analysis to uncover meaningful associations in large clinical datasets (35). Support and confidence follow the standard definitions in association rule mining, and rules are generated only when both exceed predefined minima. Based on common practice in acupuncture prescription mining and to balance rule stability with interpretability, we set support ≥20% and confidence ≥80%, consistent with prior acupuncture association-rule studies (36).

We also used the enhancement coefficient to evaluate the interdependence of item set pairs, and when the value exceeded 1, it indicated the existence of a mutual dependence relationship. The final results were analyzed in the form of a covariance matrix heat map by HemI 1.0.3.7^1^ software. After drawing the network analysis graph based on the results of the acupoint correlation analysis, the data were converted into vector form, and the correlated nodes and their weight values were output. The output nodes were used as representative points, and the connections between nodes represented the edge weights. By numbering and pairing the edge weights, a combination of the node list and the edge list was generated. Finally, the data were imported into Gephi 0.10.1^2^ for network visualization. We applied the Fruchterman Reingold algorithm to construct the central aggregation distribution model. To identify core acupoints within the co-occurrence network, we applied k-core decomposition, which extracts the maximal subgraph in which every node has at least k connections within the subgraph, thus capturing densely interconnected “backbone” structures rather than merely high-frequency nodes. This method has been adopted in complex-network analyses to determine core sets (e.g., maximum core layer as the core prescription acupoints) (37).

### 2.6 Experimental animals

A total of 27 male SPF-grade SD rats, aged 6 weeks and weighing 180 ± 20 g, were selected. The rats were supplied by Liaoning Changsheng Biotechnology Co., Ltd. (license number: SCXK (Liao) 2020-0001). The rats were housed in an environment with temperature of 22 ± 2 °C, humidity of 40–60%, natural light, fed with standard feed, and given free access to water. After 3 days of acclimatization, the experiment was conducted. All procedures complied with the regulations of the Ethics Committee of Changchun University of Chinese Medicine and the *Administrative Measures for Biosafety of Laboratory Animals*, and were approved by the Ethics Committee (Approval No. 2025353).

### 2.7 Grouping and modeling

Six rats were randomly selected as the control group (CTL) and housed under standard conditions. The remaining 21 rats were used to establish the FD model by the tail pinch stress combined with irregular feeding method (38). A long forceps wrapped in gauze was used to clip the distal 1/3 of the rat’s tail to induce a stress response, with continuous uninterrupted stimulation for 30 min, twice a day (with an interval of more than 3 h). Meanwhile, the rats were fasted every other day to induce irregular feeding, leading to disrupted hunger and satiety regulation. During this period, the rats could drink water freely. The modeling was continued for 14 days. Three rats after modeling were randomly selected for validation. The criteria for successful modeling were: slow weight gain, rough and yellowish fur, loose and foul-smelling feces, significantly reduced food intake, significantly decreased activity, and a significantly reduced gastric emptying rate, with no structural pathological damage in the gastric antrum and duodenal tissues. The successfully modeled rats were randomly divided into three groups of six rats each: model group (MOD), electroacupuncture group (EA), and Itopride group (ITP). The sample size was estimated based on preliminary experimental results, with the primary outcomes being the infiltration levels of Eos and MCs in duodenal tissues. Using G*Power software (version 3.1.9.2), a sample size of 6 rats per group was calculated to achieve 80% power at a 5% significance level (two-sided) (39).

### 2.8 Intervention methods

**Figure 1:**
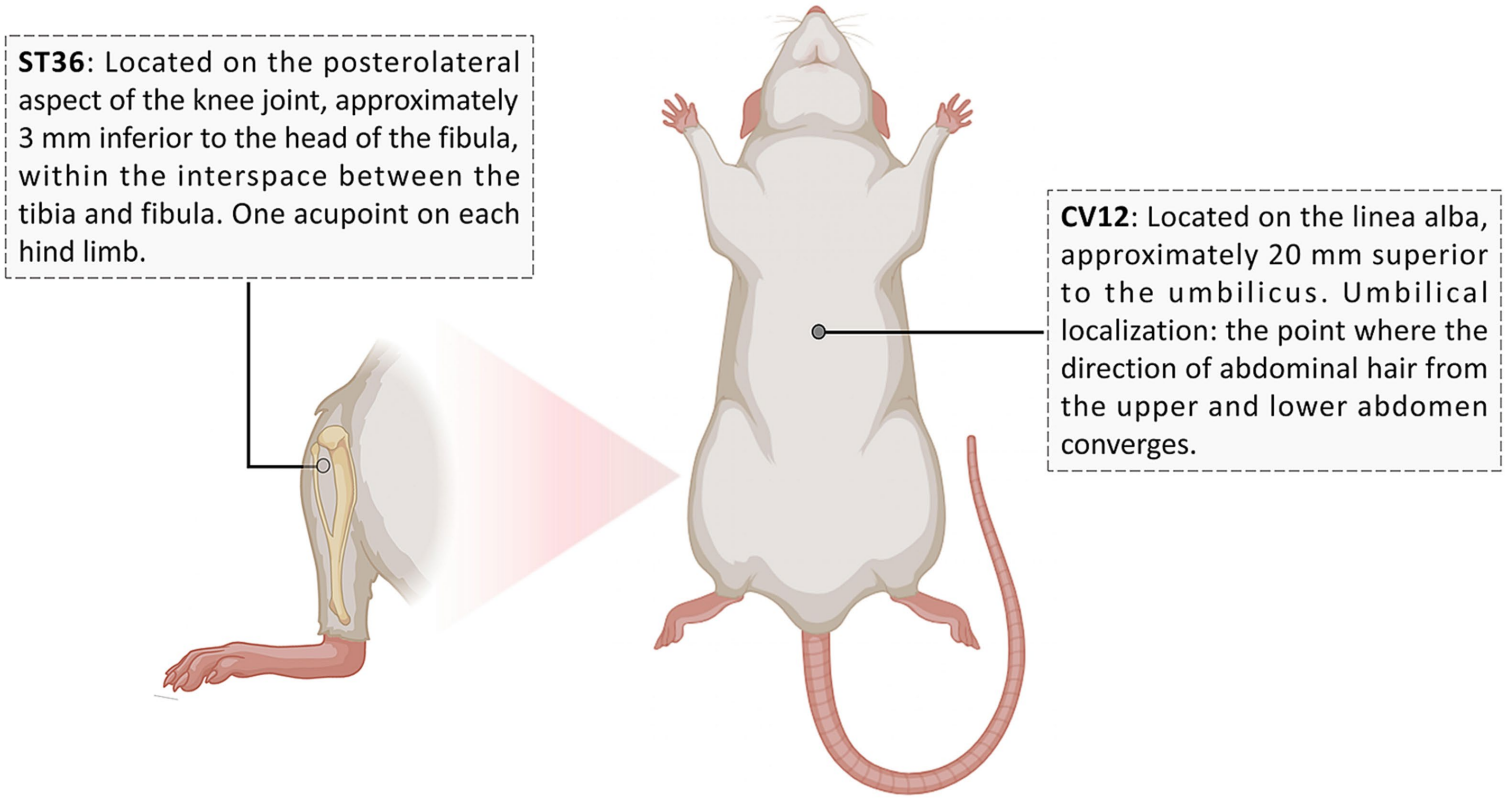
Schematic diagram illustrating the localization of ST36 and CV12 in the rat. The illustration depicts a rat in the supine position and a dorsal view of the hindlimb. Solid black dots indicate the acupoint positions. The translucent marker for ST36 denotes that the actual acupoint is situated anterior to the visible dorsal surface of the limb. Reprinted with permission from the first author JJ. Created with BioRender.com, http://biorender.com/8nkwt9k.

① Intervention method of EA: Rats were anesthetized by inhaling isoflurane (Shenzhen Reward Life Technology Co., Ltd., China, R510-22-16) at an output concentration of 3% using an anesthesia machine (Martx, United States, VMR). Then, anesthesia was maintained by 2.5% isoflurane. The rats were fixed in the supine position. CV12 and bilateral ST36 were selected [the localization method (40) is shown in Figure 1]. After local disinfection, disposable sterile acupuncture needles (Suzhou Medical Supplies Factory Co., Ltd., China, 0.25 mm × 13 mm) were used for direct puncture to a depth of 5–8 mm. After acupuncture, electroacupuncture therapeutic instrument (Suzhou Medical Supplies Factory Co., Ltd., China, SDZ-II) was connected at a frequency of 2 Hz and an intensity of 1 mA, lasting for 20 min each time, once a day for 14 days. ② Intervention method of ITP: Rats were injected by gavage with Itopride tablets (Liaoning Xinguo Pharmaceutical Co., Ltd., China, National Drug License No. H20163183) at a dose of 3 mg/kg/d once daily for 14 days. ③ CTL and MOD did not receive electroacupuncture or drug treatment. ④ To eliminate confounding variables, the MOD and ITP groups received anesthesia of the same duration and dosage as the EA group, and the EA and MOD groups were given saline gavage at the same dose as the ITP group.

After the intervention, the rats were euthanized by inhalation of an overdose of isoflurane anesthesia (5% concentration, maintained for at least 5 min). Death was confirmed by the absence of heartbeat and corneal reflex. Immediately thereafter, the abdominal cavity was opened by making an incision along the mid-abdominal line. The duodenal tissue approximately 2 cm distal to the pylorus was collected and immersed in a 4% paraformaldehyde (Beijing Solarbio Science & Technology Co., Ltd., China, P111) for fixation.

### 2.9 Observation indicators

#### 2.9.1 Monitoring body weight and food intake

Rats were weighed daily at 16:00, and the body weight changes were recorded. The rats’ mental state, fur color, activity level, food and water intake, and defecation were observed and recorded. The 3 h food intake test was conducted before and after the intervention, respectively. The rats were fasted for 12 h with free access to water, then 100 g of feed was placed in each cage. After 3 h, the remaining feed was immediately removed and weighed (W). The food intake per cage (g) = 100 − W.

#### 2.9.2 Assessing gastric emptying using methyl orange gavage

Rats were administered 3 mL of 0.1% methyl orange intragastrically (Shanghai Macklin Biochemical Co., Ltd., China, M812775). After 20 min, the rats were anesthetized and euthanized. The entire stomach was removed from the pylorus and cut open along the greater curvature. The residual pigment in the stomach was fully dissolved in 10 mL of purified water, with the pH adjusted to 6.0–6.5 using 5% sodium bicarbonate. The solution was then centrifuged at 3000 rpm for 5 min using a centrifuge (Hunan Kecheng Instrument Equipment Co., Ltd., China, H1-16KR), and the supernatant was collected. For reference, 3 mL of 0.1% methyl orange was mixed with 7 mL of purified water. The reagents were added to a 96-well plate, 50 μL per well. The optical density (OD) of methyl orange in each well was measured at a wavelength of 450 nm using a microplate reader (Thermo Scientific, United States, Multiskan FC). Gastric emptying rate (%) = [1 − (OD of gastric methyl orange/OD of reference methyl orange)] × 100%.

#### 2.9.3 Evaluating duodenal pathology by H&E staining

The duodenal tissues of rats in each group were harvested, rinsed with 0.9% NaCl solution. They were then fixed in 4% paraformaldehyde for 48 h. The tissues were then dehydrated with gradient ethanol, cleared with xylene (Sinopharm Chemical Reagent Co., Ltd., China, 10,023,418), and impregnated with paraffin (Sinopharm Chemical Reagent Co., Ltd., China, 69,019,361). Finally, they were embedded in paraffin. Sections were sliced to 4 μm thickness using a microtome (Shanghai Leica Instrument Co., Ltd., China, Leica RM 2016), dewaxed with xylene and gradient ethanol, hydrated and rinsed with distilled water. The sections were stained with hematoxylin (Sigma, United States, H9627) for 5 min, followed by bluing. After rinsing in 95% ethanol, we stained the sections with 1% alcohol-soluble eosin (Sinopharm Chemical Reagent Co., Ltd., China, 71,014,460) for 2 min. After further dehydration and xylene clearing, the sections were mounted with neutral gum (Nanchang Yulu Experimental Equipment Co., Ltd., China, 20,200,237) and then examined and photographed using a Nikon microscope (Nikon, Japan, Nikon Fi3).

#### 2.9.4 Staining observation of Eos and MCs in duodenal

The basic steps were the same as those for H&E staining. Tissue sections were first stained with hematoxylin for 5 min and then blued. For Eos detection, the sections were subsequently stained with chromotrope (TCI, Japan, C0547) for 5–10 min and rinsed under running water. After dehydration, clearing, and mounting, the Eos-stained sections were observed under a microscope. Similarly, for the toluidine blue staining used to observe MCs, sections underwent routine dewaxing and hydration, followed by staining with 1% toluidine blue (Sinopharm Chemical Reagent Co., Ltd., China, 71,041,284) for 5 min, and were then rinsed. The observation methods remained consistent.

#### 2.9.5 Determining Claudin-1 and Occludin expression by IF staining

Following dewaxing and hydration, tissue sections were subjected to high-temperature antigen retrieval in retrieval solution (100 °C, 15 min). After washing with PBS, the sections were blocked with normal goat serum (Wuhan Boster Biological Technology Co., Ltd., China, AR1009) at room temperature for 30 min and subsequently incubated with primary antibodies at 4 °C overnight. After another PBS wash, the sections were incubated with corresponding fluorescent secondary antibodies at 37 °C for 1 h in the dark. After washing with PBS to remove excess secondary antibody, the cell nuclei were counterstained with DAPI (Beyotime Biotechnology Co., Ltd., China, C1002). Finally, the sections were mounted with an anti-fade mounting medium (Southern Biotech, United States, 0100-01) and observed under a fluorescence microscope. The dilution ratios of primary antibodies were as follows: Claudin-1 (Proteintech, United States, 13050-1-AP) at 1:500 and Occludin (Affinity, United States, BF8330) at 1:200. For the secondary antibodies, Cy3-conjugated goat anti-rabbit IgG (Thermo Fisher Scientific, United States, A10520) and Cy3-conjugated goat anti-mouse IgG (Thermo Fisher Scientific, United States, A10521) were both used at a dilution of 1:400.

### 2.10 Statistics and analysis

Statistical analysis and graphing were performed using SPSS 26.0 and GraphPad Prism 10.0. Data were presented as the mean ± standard deviation. The normality of the data was assessed using the Shapiro–Wilk test, and the homogeneity of variances was verified by Levene’s test. For multiple-group comparisons, one-way analysis of variance (ANOVA) was employed, followed by Fisher’s Least Significant Difference (LSD) test for comparisons between two groups. A *p*-value of less than 0.05 was considered statistically significant.

## 3 Results

### 3.1 Data mining results

#### 3.1.1 Literature search results and assessment of bias risk

**Figure 2:**
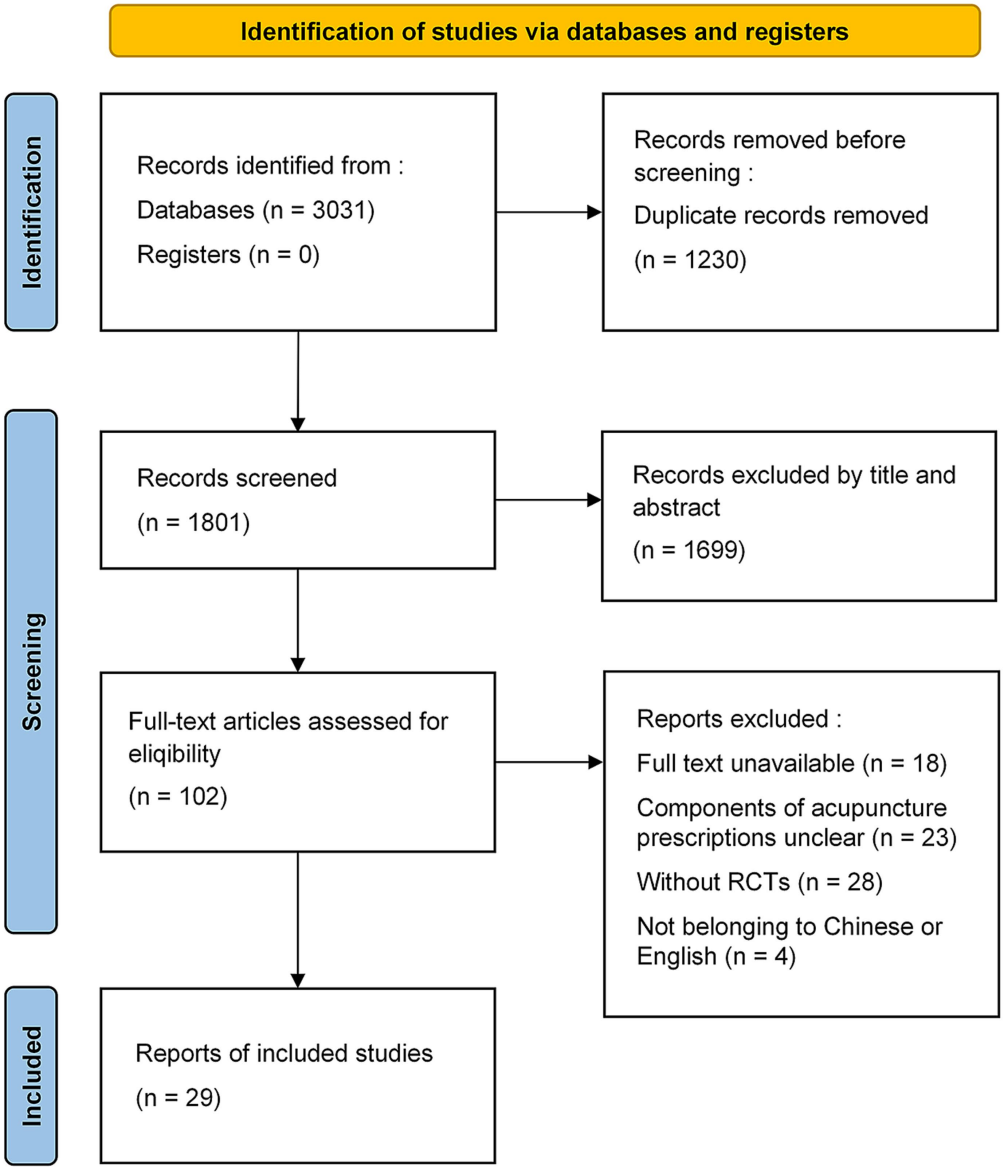
Flow diagram for the selection of articles. Adapted with permission from Page et al. (73), licensed under (CC BY 4.0.).

The literature search yielded 3,031 potentially relevant articles. Endnote X8 software was used to exclude 1,230 duplicate studies. Titles and abstracts of the remaining 1,801 studies were screened, and 102 potentially eligible studies were identified. Finally, after full-text evaluation, 29 articles were included. The detailed screening process is illustrated in Figure 2.

Among them, 25 RCTs reported complete and extractable acupoint prescriptions and were therefore included in the prescription data mining analyses; the remaining four were excluded from mining due to duplicate acupuncture prescriptions. Overall, several domains remained predominantly unclear risk due to limited reporting, and we therefore interpret the evidence base as having mainly unclear risk of bias.

**Figure 3:**
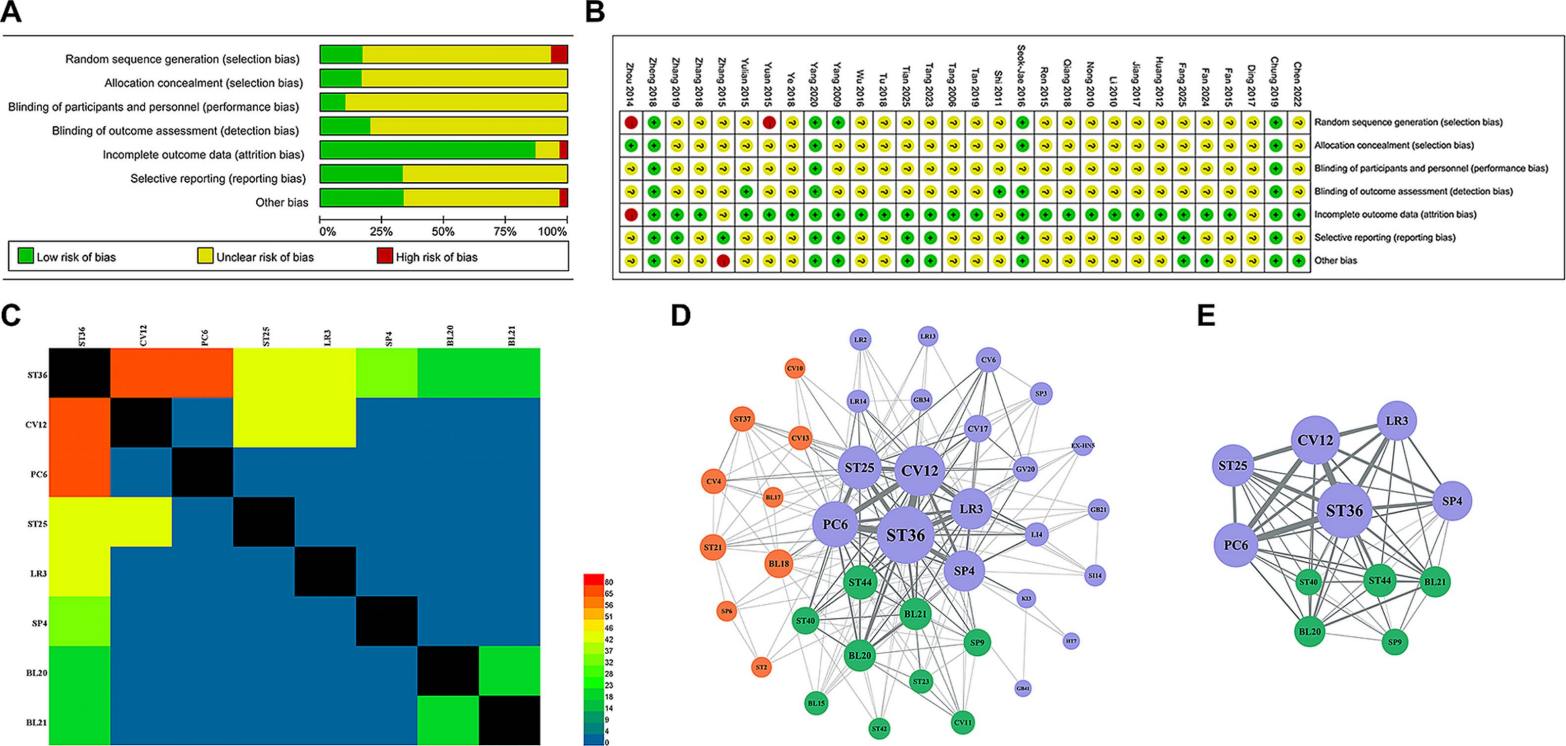
Results of data mining. **(A)** Risk of bias graph. **(B)** Risk of bias summary. **(C)** Cooccurrence matrix of acupoints. **(D)** Acupoints correlation charts. **(E)** Core acupoints chart.

Figures 3A,B present the bias assessment of the included literature. Of the 29 articles reviewed, 2 failed to adequately describe their randomization procedures, potentially introducing bias, while 5 articles reported allocation concealment, and the remainder did not specify this aspect. Only 3 articles clearly indicated the use of blinding.

#### 3.1.2 Acupoint usage frequency and combination rules

Statistics showed that a total of 42 acupoints were involved in 25 articles, used a total of 260 times (Table 1). The most frequently used acupoint was ST36. Subsequently, we summarized the compatibility rules of acupoints in 25 prescriptions (Table 2), finding that the combination of ST36 and CV12 had the highest support rate at 69.57%. This finding aligns with the co-occurrence matrix in Figure 3C, where ST36 and CV12 also showed the highest combination frequency.

**Table 1:** Frequency of acupoints in acupuncture for FD.

Number	Acupoints	Frequency	Support (%)
1	ST36	41	17.37
2	CV12	32	13.56
3	PC6	30	12.71
4	LR3	20	8.47
5	ST25	19	8.05
6	SP4	15	6.36
7	BL20	9	3.81
8	BL21	8	3.23
9	ST44	8	3.23
10	ST34	6	2.54
11	SP9	5	2.12
12	LI4	5	2.02
13	KI3	4	1.61
14	CV17	4	1.61
15	ST40	4	1.61
16	GV20	3	1.21
17	CV6	3	1.21
18	SP6	3	1.21
19	LR14	3	1.21
20	CV4	4	1.69
21	CV13	3	1.27
22	BL18	3	1.27
23	GB34	3	1.27
24	ST42	2	0.81
25	ST21	2	0.81
26	CV11	2	0.81
27	GB41	2	0.81
28	CV10	2	0.81
29	HT7	2	0.81
30	ST23	2	0.81
31	SP3	1	0.40
32	ST2	1	0.40
33	BL17	1	0.40
34	ST37	1	0.40
35	LR13	1	0.40
36	EX-HN5	1	0.40
37	BL62	1	0.40
38	KI6	1	0.40
39	BL15	1	0.40
40	SI14	1	0.40
41	GB21	1	0.40
42	LR2	1	0.40
Total	–	260	100

**Table 2:** Association rules of acupoints for FD treatment.

Number	Acupoints	Support (%)	Confidence (%)	Expected confidence (%)	Lift
1	ST36, CV12	69.57	90.63	89.13	1.02
2	CV12, PC6	65.22	80.00	69.57	1.15
3	ST36, PC6	65.22	93.33	89.13	1.05
4	ST36, PC6, CV12	52.17	95.83	89.13	1.08
5	CV12, LR3	43.48	80.00	69.57	1.15
6	ST36, LR3	43.48	95.00	89.13	1.07
7	CV12, ST25	41.30	100.00	69.57	1.44
8	ST36, ST25	41.30	89.47	89.13	1.00
9	PC6, ST25, ST36	36.96	82.35	65.22	1.26
10	CV12, ST25, ST36	36.96	100.00	69.57	1.44
11	ST36, LR3, CV12	34.78	93.75	89.13	1.05
12	ST36, SP4	32.61	93.33	89.13	1.05
13	CV12, ST25, PC6	30.43	100.00	69.57	1.44
14	ST36, ST25, PC6	30.43	100.00	89.13	1.12
15	CV12, LR3, PC6	26.09	91.67	69.57	1.32
16	ST36, LR3, PC6	26.09	100.00	89.13	1.12
17	ST36, SP4, CV12	21.74	100.00	89.13	1.12

#### 3.1.3 Network analysis of acupoints

We further evaluated whether the acupoint network exhibited a small-world structure, which is typically characterized by relatively high clustering among nodes and short average path lengths compared with a random network (41). In this context, a small-world-like topology suggests that clinical prescriptions are organized around tightly connected acupoint clusters linked by a small number of central nodes, rather than being assembled randomly.

The correlation analysis of the data mining results based on the Apriori algorithm revealed a complex network of acupoint associations, with 39 nodes and 213 edge weights. Gephi was employed to model these network associations, and ultimately revealed three distinct association types (Table 3), represented by purple, green, and orange, respectively (Figure 3D). The network model exhibited an average path length of 1.722 and a clustering coefficient of 0.82, resulting in a ratio (L/C) of 2.1. This ratio is significantly lower than that of a random network (L/C = 4.159/0.067 ≈ 62.075) under the same conditions. This result suggests that the selection of acupoints for treating FD is not a random combination, but rather a purposeful arrangement that closely connects other acupoints through core acupoints.

**Table 3:** Division of network associations in acupuncture for FD.

Cluster	Acupoint (degree value)
1	ST36(37), CV12(31), PC6(27), ST25(25), LR3(23), SP4(23), CV17(11), CV6(9), GV20(9), LI4(8), SP3(7), LR14(7), SI14(6), GB21(6), GB34(6), LR2(6) LR13(5), EX-HN5(5), KT3(4), GB41(2), HT7(2)
2	ST44(17), BL20(15), BL21(15), ST40(11), SP9(11), BL15(8), ST23(8), CV11(8), ST42(6)
3	BL18(12), ST21(10), CV4(9), ST37(9), CV13(8), ST2(5), SP6(5), CV10(5), BL17(5)

Next, we explored the core acupoint prescriptions for acupuncture treatment of FD. We determined a total of 11 core acupoints (Figure 3E). Further analysis of the network properties showed that ST36 and CV12 occupy central positions within the acupoint network. Specifically, ST36 and CV12 exhibited the highest degree, betweenness centrality, closeness centrality, and eigenvector centrality among the identified acupoints (Table 4). These metrics indicate that ST36 and CV12 are the most interconnected acupoints, serve as critical “bridge” points within the network, are in close proximity to all other acupoints, and have the strongest connections to other influential acupoints. Collectively, these findings suggest that ST36 and CV12 are the most prominent acupoints for the treatment of FD, forming the foundation upon which other acupoints are selected and applied.

**Table 4:** Analysis of core acupoints for FD treated with acupuncture.

Acupoints	Degree	Betweenness centrality	Closeness centrality	Eigenvector centrality
ST36	37	213.821861	0.974359	1
CV12	31	91.61115	0.844444	0.923906
PC6	27	66.83139	0.77551	0.828638
ST25	25	45.90043	0.745098	0.811688
LR3	23	34.60357	0.716981	0.76529
SP4	23	40.62424	0.716981	0.752505
ST44	17	8.337338	0.644068	0.666615
ST40	11	0.924242	0.584615	0.509238
BL20	15	5.189719	0.622951	0.606129
BL21	15	5.189719	0.622951	0.606129
SP9	11	1.333333	0.584615	0.486224

In conclusion, the results demonstrated that ST36 is the most frequently utilized acupoint for FD treatment, with ST36 and CV12 being the most common combination. Network association analysis further revealed that ST36 and CV12 are the most closely interconnected acupoints, occupying central positions. Based on these findings, we infer that their combination is crucial to the efficacy of acupuncture in treating FD and forms the core of the acupoint prescription. Consequently, we conducted animal experiments to validate this finding.

### 3.2 Experimental verification results

#### 3.2.1 Improved digestive function and nutritional status

**Figure 4:**
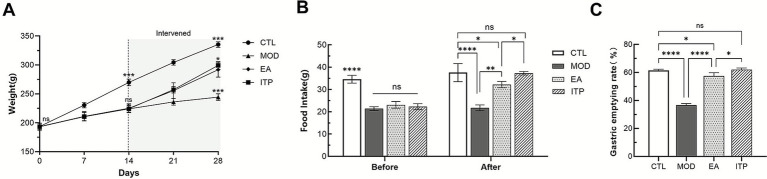
Effects of electroacupuncture intervention on body weight, 3-hour food intake, and gastric emptying rate in functional dyspepsia rats. CTL, control group; MOD, model group; EA, electroacupuncture group; ITP, itopride group. **(A)** Body weight changes of rats in each group at different stages. **(B)** Comparison of 3-hour food intake before and after intervention in each group. **(C)** Gastric emptying rate after intervention compared among groups by measuring the optical density (OD) of methyl orange. Data are presented as mean ± SD, and error bars represent standard deviation. *P* values were calculated by one-way ANOVA followed by Fisher’s LSD test. * *P* < 0.05, ** *P* < 0.01, *** *P* < 0.001, **** *P* < 0.0001, ns, not significant.

FD commonly presents with obvious symptoms such as reduced appetite, weight loss, and insufficient gastric motility. Electroacupuncture has shown efficacy in alleviating these symptoms. In this study, we recorded the rats’ daily body weight and plotted the line graph of changes over time. As depicted in Figure 4A, compared to the MOD group, rats in the EA group exhibited a significant increase in body weight following the intervention (*p* < 0.001). We also measured the food intake of rats in each group within a 3 h period, with results shown in Figure 4B. After the intervention, the EA group showed a significant rise in food consumption relative to the MOD group (*p* < 0.01). Furthermore, we assessed the gastric emptying rate by measuring the OD of methyl orange in gastric contents of rats. Figure 4C illustrates that the EA group had a significantly enhanced gastric emptying rate compared to the MOD group (*p* < 0.0001). This finding suggests that electroacupuncture at ST36 and CV12 effectively improves the digestive function and nutritional status in FD rats, although its effect is less pronounced than that of ITP, it still plays a valuable role.

#### 3.2.2 Alleviated duodenal low-grade inflammation

**Figure 5:**
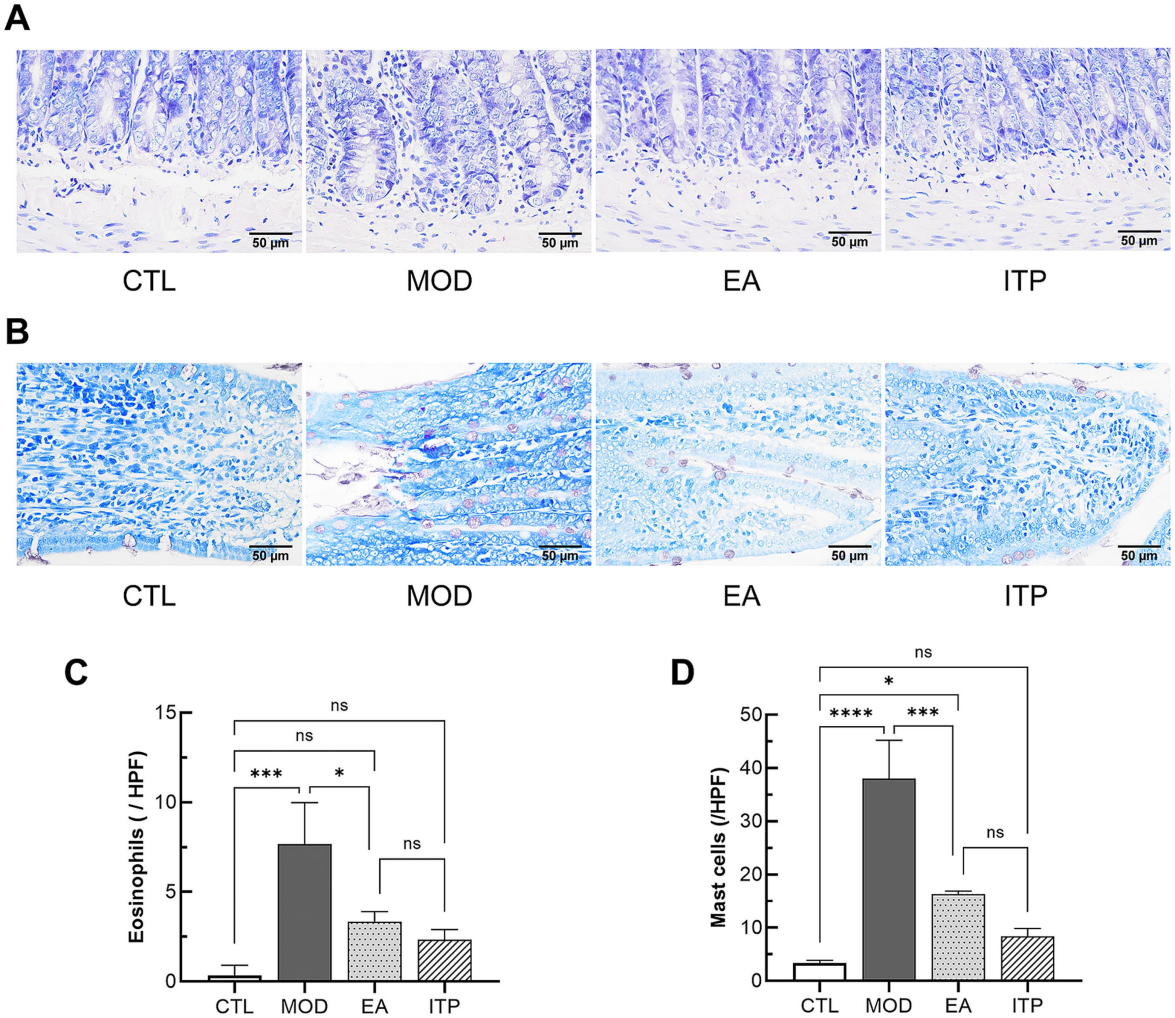
Staining observations of eosinophils (Eos) and mast cells (MCs) in the duodenum of functional dyspepsia rats regulated by electroacupuncture. **(A)** Representative Eos staining images of duodenal tissues from each group (400× magnification). The cell nuclei appear blue, and the eosinophilic granules appear red. **(B)** Representative toluidine blue staining images of duodenal tissues from each group (400× magnification). MCs appear blue-violet. **(C)** The average number of Eos per field. **(D)** The average number of MCs per field. Data are presented as mean ± SD, and error bars represent the standard deviation. *P* values were calculated by one-way ANOVA followed by Fisher’s LSD test. * *P* < 0.05, *** *P* < 0.001, **** *P* < 0.0001; ns, not significant.

Subsequent experiments mainly focused on the pathogenesis of duodenal low-grade inflammation and increased mucosal permeability in FD. The primary indicator of this inflammation is the elevated infiltration of Eos and MCs. Staining observations revealed significant infiltration of Eos and MCs in the duodenal tissues of the MOD group, as shown in Figures 5A,B. Compared with the MOD group, the number of Eos was significantly decreased in the EA group (*p* < 0.05) and showed no significant difference compared to either the CTL or ITP groups (*p* > 0.05) (Figure 5C). Similarly, after electroacupuncture intervention, the number of MCs in the EA group was also markedly reduced compared to the MOD group (*p* < 0.001), with no significant difference observed when compared to the ITP group (*p* > 0.05) (Figure 5D). These findings demonstrate that electroacupuncture at ST36 and CV12 effectively ameliorates the infiltration of both Eos and MCs in the duodenum of FD rats, thereby alleviating low-grade inflammation.

#### 3.2.3 Restored the duodenal mucosal barrier

**Figure 6:**
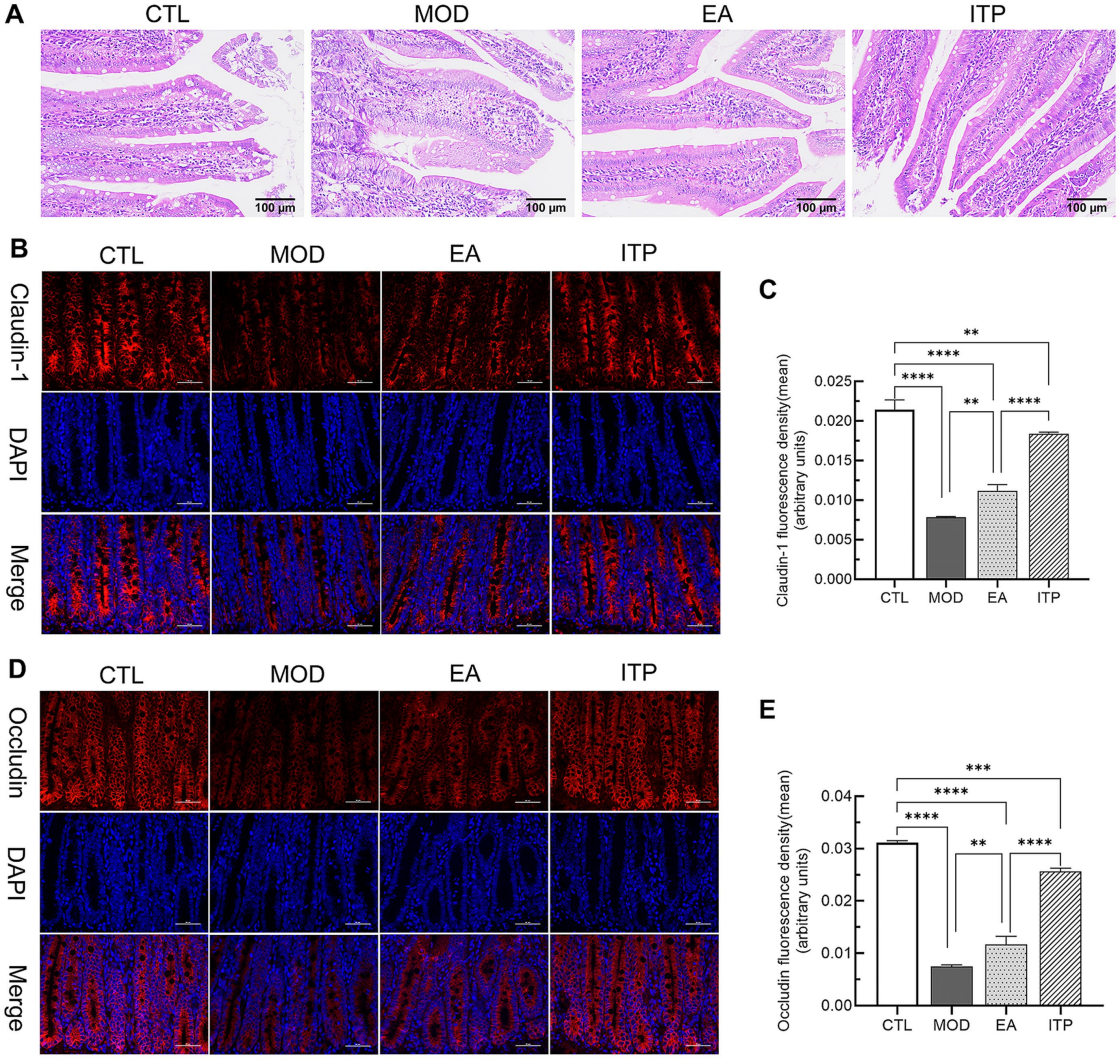
Pathological observation and immunofluorescence (IF) analysis of tight junction proteins (TJs) in the duodenal mucosal barrier of functional dyspepsia rats following electroacupuncture treatment. **(A)** Representative H&E staining images of duodenal tissues from each group (200× magnification). **(B)** IF staining images showing the expression of Claudin-1 (red) in the duodenal mucosal barrier. Nuclei were counterstained with DAPI (blue) (400× magnification). **(C)** Quantitative analysis of the average fluorescence intensity of Claudin-1. **(D)** IF staining images showing the expression of Occludin (red) in the duodenal mucosal barrier. Nuclei were counterstained with DAPI (blue) (400× magnification). **(E)** Quantitative analysis of the average fluorescence intensity of Occludin. Data are presented as mean ± SD, and error bars represent standard deviation. *P* values were calculated by one-way ANOVA followed by Fisher’s LSD test. ** *P* < 0.01, *** *P* < 0.001, **** *P* < 0.0001.

The degree of damage to the duodenal mucosal barrier can be assessed through histological morphology and the expression level and distribution of TJs between epithelial cells. This study utilized these indicators to investigate the reparative effects of electroacupuncture. H&E staining (Figure 6A) revealed that the duodenal tissue of the MOD group exhibited slightly disordered arrangement of intestinal villi, with fusion, blunting, and shedding of some villi. In contrast, the EA group showed a more intact mucosal structure with neatly arranged villi.

Next, IF staining was used to observe the expression of TJs within the mucosal barrier. Claudin-1 and Occludin are critical TJs that regulate intestinal mucosal permeability. As shown in Figures 6B–E, fluorescence signals for Claudin-1 and Occludin were significantly reduced in the duodenal tissues of the MOD group compared to CTL (*p* < 0.0001). In contrast, the expression levels of these proteins were markedly elevated in the duodenum of the EA group relative to the MOD group (*p* < 0.01). These findings suggest that electroacupuncture at ST36 and CV12 can effectively restore the compromised duodenal mucosal barrier in FD rats. This restoration contributes to stabilizing the duodenal microenvironment and alleviating FD-related symptoms.

In conclusion, these experiments employed a range of histological techniques to observe the effects of electroacupuncture at ST36 and CV12 in FD rats. The results demonstrated that this intervention not only improved digestive function in FD rats, but also regulated duodenal low-grade inflammation, attenuated the infiltration of Eos and MCs, and effectively repaired the damaged duodenal mucosal barrier, thereby ameliorating the disrupted duodenal microenvironment in FD. These findings suggest that the combined use of ST36 and CV12 represents a core acupuncture prescription with excellent therapeutic potential for treating FD.

## 4 Discussion

### 4.1 Research findings: the core acupoint combination for FD

Although acupuncture therapy demonstrates unique advantages in alleviating FD clinical symptoms and reducing recurrence rates (42), the substantial individual variation in acupoint prescriptions severely compromises the reproducibility of clinical practice. A major challenge in current research lies in achieving standardization in prescription application while adhering to the individualized principles of Chinese medicine. Therefore, this study aims to identify a high-frequency acupoint combination for treating FD, thereby establishing a foundational therapeutic framework. This framework is intended to serve as a reference for enhancing the stability and reproducibility of acupuncture efficacy.

We conducted a systematic search of 8 databases, and after screening, included 21 acupoint prescriptions in our analysis. Data mining revealed that ST36 was the most frequently used acupoint and was often combined with CV12 to form the predominant acupoint combination. Moreover, network association analysis highlighted the central role of ST36 and CV12 as critical “bridge” nodes within the acupoint prescription network, indicating their prominent therapeutic effects. By integrating multiple analytical methods, this study clarified the central importance of ST36 and CV12 in acupoint prescriptions for treating FD.

Subsequently, we conducted confirmatory animal experiments. We performed a series of histological analyses, including Eos staining, toluidine blue staining, H&E staining, and IF staining on duodenal tissues of FD model rats. The results showed that compared to the MOD group, the EA group, which received electroacupuncture at ST36 and CV12, exhibited a significant reduction in infiltration of Eos and MCs in the duodenal tissues. Additionally, electroacupuncture intervention notably increased the expression levels of Claudin-1 and Occludin, suggesting that the intestinal mucosal permeability was effectively improved. These results confirm that electroacupuncture at ST36 and CV12 therapeutically improves low-grade inflammation and barrier damage in the FD duodenum, thereby reconstructing duodenal microenvironment homeostasis. Next, we explored the underlying physiological mechanisms of ST36 and CV12 as the core acupoints for therapeutic effects.

### 4.2 Converging evidence: mechanistic insights into ST36 and CV12 from previous studies

Existing evidence suggests that electroacupuncture at ST36 activates vagal activity, inhibits sympathetic nerve activity, and effectively improves delayed gastric emptying in FD rats (43). It also alleviates pathological changes in interstitial cells of Cajal and restores gastrointestinal motility (44–46). Furthermore, the phenomenon of MC degranulation in the gastric mucosa of FD rats can be significantly ameliorated by electroacupuncture at ST36, thereby reducing visceral hypersensitivity (47). Notably, ST36 also has a regulatory effect on low-grade inflammation and barrier function in the duodenum, as it can inhibit the major basic protein of Eos and restore the expression level of Occludin-1 (48). These findings align closely with our study’s results, further confirming the crucial role of ST36 in restoring duodenal pathological changes in FD.

On the other hand, CV12, an important local acupoint located in the gastric body-surface projection area, demonstrates significant efficacy in relieving typical FD symptoms such as stomachache and bloating. Research indicates that CV12 can modulate the signal transduction of gastrointestinal neurotransmitters, reduce mitochondrial damage, and inhibit cell apoptosis, thereby enhancing the activity of digestive enzymes and improving the gastrointestinal digestive function in FD rats (49). Furthermore, regarding the pathological changes in the duodenum of FD, electroacupuncture at CV12 has also been shown to upregulate the expression of tight junction proteins, such as Claudin-3 and ZO-1 (48).

Both ST36 and CV12 are important in treating FD, yet their combination produces better therapeutic effects, may be synergistic. A study showed that electroacupuncture at CV12 alone promotes rapid gastric emptying, and combining it with ST36 significantly improves efficacy compared to single-point stimulation (50). Another study, highly relevant to our experimental design, found that electroacupuncture at ST36 and CV12 significantly reduces the number of MCs in the duodenum of FD rats (51). This provides compelling preliminary evidence supporting the results of the subsequent animal experiments in our study.

### 4.3 Duodenal pathology: an emerging therapeutic target for FD

The traditional view that FD is a disease without organic lesions and primarily affects the stomach, has been challenged by recent advancements. Emerging evidence from duodenal endoscopy has revealed pathological changes in FD patients, including duodenal mucosal barrier damage and increased Eos infiltration in biopsy samples (52, 53). These findings suggest that the duodenum may be a crucial site for the generation of FD symptoms (54–56). Various previously proposed pathogenic mechanisms may be related to disorders in the duodenal internal environment (57). Therefore, the duodenum has recently become a focus of FD pathological research and a potential therapeutic target (58, 59). Clinical observations have confirmed that interventions targeting duodenal Eos infiltration can alleviate FD symptoms (60, 61). However, there are currently few research reports on the regulation of duodenal pathological changes in FD by acupuncture. Therefore, this study used these parameters as observation indicators in animal experiments to evaluate the efficacy of the core acupoint combination and to explore potential acupuncture targets for FD treatment. The experimental results supported our hypothesis, as electroacupuncture at ST36 and CV12 significantly alleviated low-grade inflammation and barrier damage in the duodenum of FD model rats.

The intestinal mucosal barrier is essential for maintaining intestinal microenvironment homeostasis (62, 63). Its integrity primarily relies on tight junction structures located at the apical side of intestinal epithelial cells (64). Composed of proteins such as Occludin, Claudin family proteins, and ZO proteins, TJs regulate mucosal permeability (65). A significant reduction in TJ expression increases duodenal mucosal permeability (66), allowing luminal contents to penetrate the barrier and reach the lamina propria, thus triggering an intestinal immune response (67). The resulting inflammatory factors can further compromise the mucosal barrier, creating a vicious cycle. The findings of this study align with the above information. Experimental results indicate significant infiltration of Eos and MCs in the duodenal tissue of FD rats, accompanied by a marked reduction in Claudin-1 and Occludin expression. Notably, increased duodenal permeability is not only associated with visceral pain sensitization but may also impact gastric emptying (68, 69). Furthermore, the activation of Eos and MCs can release various neuroactive and inflammatory mediators, leading to local nerve stimulation and abnormal smooth muscle contraction, which may ultimately manifest as symptoms such as abdominal pain and bloating (70–72). Thus, this study not only validates the efficacy of the core acupoint combination but also provides experimental evidence for the pathogenesis of FD at the mechanistic level.

### 4.4 Study limitations and future perspectives

We acknowledge several limitations in the present study. First, the literature inclusion criteria for data mining were restricted to RCTs published in English or Chinese, which may have introduced selection and language biases, thereby potentially compromising the comprehensiveness of the clinical evidence. Second, due to practical constraints, both the intervention administrators and outcome assessors were not blinded to group allocation, which could have introduced potential bias into the results. Finally, the current experimental design did not specifically verify the specific effects, relative superiority, or synergistic interactions of this acupoint combination. Future research should focus on designing more rigorous and diversified controlled experiments (e.g., including sham-acupoint, single-acupoint, or alternative combination groups) to clarify the therapeutic advantages of this core acupoint combination. Furthermore, translating these fundamental findings into applicable and verifiable treatment protocols for clinical practice represents a critical direction for subsequent work.

## 5 Conclusion

This study indicates ST36 and CV12 as the core acupoint combination for the acupuncture treatment of FD. Animal experiments further demonstrate that electroacupuncture stimulation of these acupoints significantly alleviates duodenal low-grade inflammation and repairs mucosal barrier damage in FD rats, thereby supporting the biological plausibility and treatment relevance of this combination. These findings not only establish the core framework for acupoint prescription in FD treatment but also supplement the mechanism of action of acupuncture in treating FD.

## Data Availability

The original contributions presented in the study are included in the article/Supplementary material, further inquiries can be directed to the corresponding authors.

## Footnotes

1
http://hemi.biocuckoo.org/

2
https://gephi.org/
