# Supplementary material for: From data mining to experimental validation: ST36 and CV12 as core acupoint combination for acupuncture in functional dyspepsia
Source: Front Med (Lausanne). 2026 Mar 10;13:1728742. doi: 10.3389/fmed.2026.1728742 (PMC13011851; doi:10.3389/fmed.2026.1728742)
Supplement: Supplementary file 1 [file Supplementary_file_1.docx]

**Supplementary Material 1. Search strategies of each database**

The following database will be searched from inception to 30, June, 2025.

**Part 1：Search term**

**1.Acupuncture**：

**MESH**：Acupuncture OR Acupuncture Therapy OR Acupuncture Points

**TI OR AB**: acupuncture or acupuncture needle or needling or body acupuncture or acupressure or acupoint* or manual acupuncture or MA or electroacupuncture or electro-acupuncture or EA or laser acupuncture or warm needling or meridian*

**2.Disease：**

**MESH**: Dyspepsia

**TI OR AB**: functional dyspepsia or functional gastrointestinal disorder or dyspep* or non-ulcer dyspepsia or idiopathic dyspepsia or indigestion or indigestive or NUD or FD or satiety or epigastric pain or upper GI symptom*

**3.Clinical type**：

**MESH**: clinical trials as topic or random allocation

**Publication Type**: clinical trial

**TI OR AB**: (clinical[tiab] AND trial[tiab]) or random* or trial or randomized controlled trial or controlled clinical

**Part 2 Database**

**1.Pubmed（https://pubmed.ncbi.nlm.nih.gov/）**

((((((clinical[Title/Abstract]) AND (trial[Title/Abstract])) OR ((((random*[Title/Abstract]) OR (trial[Title/Abstract])) OR (randomized controlled trial[Title/Abstract])) OR (controlled clinical[Title/Abstract]))) OR ((clinical trials as topic[MeSH Terms]) OR (random allocation[MeSH Terms]))) OR (clinical trial[Publication Type])) AND ((Dyspepsia[MeSH Terms]) OR ((((((((((((functional dyspepsia[Title/Abstract]) OR (functional gastrointestinal disorder[Title/Abstract])) OR (dyspep*[Title/Abstract])) OR (non-ulcer dyspepsia[Title/Abstract])) OR (idiopathic dyspepsia[Title/Abstract])) OR (indigestion[Title/Abstract])) OR (indigestive[Title/Abstract])) OR (NUD[Title/Abstract])) OR (FD[Title/Abstract])) OR (satiety[Title/Abstract])) OR (epigastric pain[Title/Abstract])) OR (upper GI symptom*[Title/Abstract])))) AND ((((Acupuncture[MeSH Terms]) OR (Acupuncture Therapy[MeSH Terms])) OR (Acupuncture Points[MeSH Terms])) OR ((((((((((((((acupuncture[Title/Abstract]) OR (acupuncture needle[Title/Abstract])) OR (needling[Title/Abstract])) OR (body acupuncture[Title/Abstract])) OR (acupressure[Title/Abstract])) OR (acupoint*[Title/Abstract])) OR (manual acupuncture[Title/Abstract])) OR (MA[Title/Abstract])) OR (electroacupuncture[Title/Abstract])) OR (electro-acupuncture[Title/Abstract])) OR (EA[Title/Abstract])) OR (laser acupuncture[Title/Abstract])) OR (warm needling[Title/Abstract])) OR (meridian*[Title/Abstract])))

**2.Corchrane library (https://www.cochranelibrary.com/)**

#1：MeSH descriptor: [Acupuncture] explode all trees

#2：MeSH descriptor: [Acupuncture Therapy] explode all trees

#3：MeSH descriptor: [Acupuncture Points] explode all trees

#4：#1 or #2 or #3

#5：(acupuncture or acupuncture needle or needling or body acupuncture or acupressure or acupoint* or manual acupuncture or MA or electroacupuncture or electro-acupuncture or EA or laser acupuncture or warm needling or meridian*):ti,ab,kw

#6：#4 or #5

#7：MeSH descriptor: [Dyspepsia] explode all trees

#8：(functional dyspepsia or functional gastrointestinal disorder or dyspep* or non-ulcer dyspepsia or idiopathic dyspepsia or indigestion or indigestive or NUD or FD or satiety or epigastric pain or upper GI symptom*):ti,ab,kw

#9：#7 or #8

#10：MeSH descriptor: [Clinical Trials as Topic] explode all trees

#11：MeSH descriptor: [Random Allocation] explode all trees

#12：#10 or #11

#13：(clinical trial):pt

#14：(clinical):ti,ab,kw

#15：(trial):ti,ab,kw

#16：#14 or #15

#17：(random* or trial or randomized controlled trial or controlled clinical):ti,ab,kw

#18：#12 or #13 or #16 or #17

#19：#6 and #9 and #18

**3.Embase (https://www.embase.com/)**

#1：MeSH descriptor: [Acupuncture] explode all trees

#2：MeSH descriptor: [Acupuncture Therapy] explode all trees

#3：MeSH descriptor: [Acupuncture Points] explode all trees

#4：#1 or #2 or #3

#5：(acupuncture or 'acupuncture needle' or needling or 'body acupuncture' or acupressure or acupoint* or 'manual acupuncture' or MA or electroacupuncture or electro-acupuncture or EA or 'laser acupuncture' or 'warm needling' or meridian*)

#6：#4 or #5

#7：MeSH descriptor: [Dyspepsia] explode all trees

#8：('functional dyspepsia' or 'functional gastrointestinal disorder' or dyspep* or 'non-ulcer dyspepsia' or 'idiopathic dyspepsia' or indigestion or indigestive or NUD or FD or satiety or 'epigastric pain' or 'upper GI symptom*'):ti,ab,kw

#9：#7 or #8

#10：MeSH descriptor: [Clinical Trials as Topic] explode all trees

#11：MeSH descriptor: [Random Allocation] explode all trees

#12：#10 or #11

#13：('clinical trial'):pt

#14：(clinical):ti,ab,kw

#15：(trial):ti,ab,kw

#16：#14 or #15

#17：(random* or trial or 'randomized controlled trial' or 'controlled clinical'):ti,ab,kw

#18：#12 or #13 or #16 or #17

#19：#6 and #9 and #18

**4.Web of Science (https://webofscience.clarivate.cn/)**

#1 'acupuncture'/exp

#2 'acupuncture therapy'/exp

#3 'acupuncture points'/exp

#4 acupuncture:ta,ab,kw OR 'acupuncture needle':ta,ab,kw OR needling:ta,ab,kw OR 'body acupuncture':ta,ab,kw OR acupressure:ta,ab,kw OR acupoint*:ta,ab,kw OR 'manual acupuncture':ta,ab,kw OR ma:ta,ab,kw OR electroacupuncture:ta,ab,kw OR 'electro acupuncture':ta,ab,kw OR ea:ta,ab,kw OR 'laser acupuncture':ta,ab,kw OR 'warm needling':ta,ab,kw OR meridian*:ta,ab,kw

#5 #1 OR #2 OR #3

#6 #4 OR #5

#7 'dyspepsia'/exp

#8 'functional dyspepsia':ta,ab,kw OR 'functional gastrointestinal disorder':ta,ab,kw OR dyspep*:ta,ab,kw OR 'non-ulcer dyspepsia':ta,ab,kw OR 'idiopathic dyspepsia':ta,ab,kw OR indigestion:ta,ab,kw OR indigestive:ta,ab,kw OR nud:ta,ab,kw OR fd:ta,ab,kw OR satiety:ta,ab,kw OR 'epigastric pain':ta,ab,kw OR 'upper gi symptom*':ta,ab,kw

#9 #7 OR #8

#10 'clinical trials as topic'/exp

#11 'random allocation'/exp

#12 #10 OR #11

#13 'clinical trial':it

#14 'clinical':ta,ab,kw AND 'trial':ta,ab,kw

#15 random*:ta,ab,kw OR trial:ta,ab,kw OR 'randomized controlled trial':ta,ab,kw OR 'controlled clinical':ta,ab,kw

#16 #12 OR #13 OR #14 OR #15

#17 #6 AND #9 AND #16

**5.VIP(https://www.cqvip.com/)**

((M=functional dyspepsia OR indigestion OR non-ulcer dyspepsia OR idiopathic dyspepsia) OR (R=functional dyspepsia OR indigestion OR non-ulcer dyspepsia OR idiopathic dyspepsia)) AND ((M=acupuncture OR electroacupuncture OR hand acupuncture OR filiform needle OR warm acupuncture OR laser acupuncture) OR (R=acupuncture OR electroacupuncture OR hand acupuncture OR filiform needle OR warm acupuncture OR laser acupuncture)) AND ((M=randomized OR trial OR control) OR (R=randomized OR trial OR control))

**6.Wanfang (https://www.wanfangdata.com.cn/)**

(functional dyspepsia OR indigestion OR non-ulcer dyspepsia OR idiopathic dyspepsia) AND (acupuncture OR electroacupuncture OR hand acupuncture OR filiform needle OR warm acupuncture OR laser acupuncture) AND (randomized OR trial OR control))

**7.China National Knowledge Infrastructure (CNKI)(https://www.cnki.net/)**

SU %= 'Functional Dyspepsia' OR SU %= 'Dyspepsia' OR SU %= 'Non-ulcer Dyspepsia' OR SU %= 'idiopathic dyspepsia' OR SU %= 'Burning' OR SU %= 'Anorexia' OR SU %= 'Upper Abdominal Discomfort' OR SU %= 'Upper Abdominal Pain' OR SU %= 'Bloating' OR SU %= 'Early Saturation' OR SU %= 'Belching' OR TKA %= 'Functional Dyspepsia' OR TKA %= 'Dyspepsia' OR TKA %= 'Non-ulcer Dyspepsia' OR TKA %= 'idiopathic dyspepsia' OR TKA %= 'Burning' OR TKA %= 'Anorexia' OR TKA %= 'Upper Abdominal Discomfort' OR TKA %= 'Upper Abdominal Pain' OR TKA %= 'Bloating' OR TKA %= 'Early Saturation' OR TKA %= 'Belching'

SU %= 'acupuncture' OR SU %= 'electroacupuncture' OR SU %= 'hand acupuncture' OR SU %= 'acupoint' OR SU %= 'acupuncture manipulation' OR SU %= 'filiform needle' OR SU %= 'warm acupuncture' OR SU %= 'laser acupuncture' OR TKA %= 'acupuncture' OR TKA %= 'electroacupuncture' OR TKA %= 'hand acupuncture' OR TKA %= 'acupoint' OR TKA %= 'acupuncture manipulation' OR TKA %= 'filiform needle' OR TKA %= 'warm acupuncture' OR TKA %= 'laser acupuncture'

SU %= 'randomized' OR SU %= 'trial' OR SU %= 'control' OR TKA %= 'randomized' OR TKA %= 'trial' OR TKA %= 'control'

**8.SinoMed (Chinese Biomedical Database) (https://www.sinomed.ac.cn/index.jsp)**

(("Functional Dyspepsia"[Common field: Intelligent] OR "Dyspepsia"[Common field: Intelligent] OR "Non-ulcer Dyspepsia"[Common field: Intelligent] OR "idiopathic dyspepsia"[Common field: Intelligent]) AND( "acupuncture"[Common field: Intelligent] OR "electroacupuncture"[Common field: Intelligent] OR "hand acupuncture"[Common field: Intelligent] OR "filiform needle"[Common field: Intelligent] OR "warm acupuncture"[Common field: Intelligent] OR "laser acupuncture"[Common field: Intelligent])) AND ( "randomized"[Common field: Intelligent] OR "control"[Common field: Intelligent] OR "trial"[Common field: Intelligent])
