# Supplementary material for: From data mining to experimental validation: ST36 and CV12 as core acupoint combination for acupuncture in functional dyspepsia
Source: Front Med (Lausanne). 2026 Mar 10;13:1728742. doi: 10.3389/fmed.2026.1728742 (PMC13011851; doi:10.3389/fmed.2026.1728742)
Supplement: Supplementary file 2 [file Supplementary_file_2.docx]

**Supplementary Material 2. Table 1 Frequency of acupoints in acupuncture for FD**

Table 1 Frequency of acupoints in acupuncture for FD

| Number | Acupoints | Frequency | Support (%) |
| --- | --- | --- | --- |
| 1 | ST36 | 41 | 17.37 |
| 2 | CV12 | 32 | 13.56 |
| 3 | PC6 | 30 | 12.71 |
| 4 | LR3 | 20 | 8.47 |
| 5 | ST25 | 19 | 8.05 |
| 6 | SP4 | 15 | 6.36 |
| 7 | BL20 | 9 | 3.81 |
| 8 | BL21 | 8 | 3.23 |
| 9 | ST44 | 8 | 3.23 |
| 10 | ST34 | 6 | 2.54 |
| 11 | SP9 | 5 | 2.12 |
| 12 | LI4 | 5 | 2.02 |
| 13 | KI3 | 4 | 1.61 |
| 14 | CV17 | 4 | 1.61 |
| 15 | ST40 | 4 | 1.61 |
| 16 | GV20 | 3 | 1.21 |
| 17 | CV6 | 3 | 1.21 |
| 18 | SP6 | 3 | 1.21 |
| 19 | LR14 | 3 | 1.21 |
| 20 | CV4 | 4 | 1.69 |
| 21 | CV13 | 3 | 1.27 |
| 22 | BL18 | 3 | 1.27 |
| 23 | GB34 | 3 | 1.27 |
| 24 | ST42 | 2 | 0.81 |
| 25 | ST21 | 2 | 0.81 |
| 26 | CV11 | 2 | 0.81 |
| 27 | GB41 | 2 | 0.81 |
| 28 | CV10 | 2 | 0.81 |
| 29 | HT7 | 2 | 0.81 |
| 30 | ST23 | 2 | 0.81 |
| 31 | SP3 | 1 | 0.40 |
| 32 | ST2 | 1 | 0.40 |
| 33 | BL17 | 1 | 0.40 |
| 34 | ST37 | 1 | 0.40 |
| 35 | LR13 | 1 | 0.40 |
| 36 | EX-HN5 | 1 | 0.40 |
| 37 | BL62 | 1 | 0.40 |
| 38 | KI6 | 1 | 0.40 |
| 39 | BL15 | 1 | 0.40 |
| 40 | SI14 | 1 | 0.40 |
| 41 | GB21 | 1 | 0.40 |
| 42 | LR2 | 1 | 0.40 |
| Total | — | 260 | 100 |
